# The Law: Is That All?

**Published:** 1881-01

**Authors:** C. Pearson

**Affiliations:** Washington, D. C.


### THE "LAW:" IS THIS ALL?

BY C. PEARSON, M.D., WASHINGTON, D. C.

If the law of similars is all that constitutes Homœopathy, we are disciples of Hippocrates, not of Hahnemann. A class of pseudo-homœopaths insist on engrafting the name of the latter on the crude theories of the former; and could they succeed, the death of Homœopathy would only be a matter of time, for names without principles are short-lived.

This crude kind of Homœopathy has been dead and buried for over a thousand years, why resurrect it now? The facts are plain and unmistakable. When Hahnemann first commenced to apply the law with crude drugs he found it difficult to diminish the dose sufficiently, and in his efforts to do this discovered the process of attenuation, out of which grew his theory of dynamization. In judging of his system we are not to accept as the standard the years of his experimenting for its development, the mere skeleton or model of the future structure, but rely on, and profit by, what time and experience taught him.

It is humiliating to hear a professed Hahnemannian use such language as the following: "As a Homœopathician we consider ourselves bound solely by the law of the similars, and not by the dose or by the dynamization of the remedy." If such a declaration of principles is to be accepted as the standard of *the* Homœopathy of to-day, it is not difficult to predict what the end will be.

In advocacy of the law and crude dosing, every conceivable argument has been brought to bear; even the microscope is appealed to, but like the guests at the banquet, it can not see the ghost of Banquo. It is questionable whether it is more aggravating than amusing, to see these microscopists trying to demonstrate how much *nothing* there is in the thirtieth or the C. M. potency. Will they be kind enough to weigh us out a portion of small-pox contagion, a charge of electricity, or the fragrance of a flower? No advocate of high potencies ever for one moment believed this possible. Hahnemann himself did not believe it; he says the doses he advises "are so small as to be undetectible by the senses, and by every conceivable chemical analysis." But are they to be rejected on this account, when their curative properties are increased with increased dynamization up, certainly, to the thousands? But what is the proof, and who are the witnesses in reference to this matter. No one is competent to testify who has not for years in daily practice tested both; an opinion formed without this experience must be received only as an opinion, not as knowledge. For the first ten years of my practice, my prescriptions were mainly made with medicines from the third to the twelfth, sometimes with the thirtieth. For the past twenty years they have ranged from the thirtieth to the C M very rarely now below 1 M and my experience has been that just in proportion as I have ascended the scale, in equal proportion has my mortality list diminished. And this fact, it seems to me, can only be accounted for in one of three ways: First, that with increasing years my knowledge of the Materia Medica has also increased, enabling me to select with greater certainty the truly Homœopathic remedy. While there may be some plausibility in this, it is not of itself sufficient to account for the difference.

Young practitioners usually feel more responsibility, hence, spend more time in studying their cases than older physicians, and they generally have more leisure to do this. Then it may be urged that as we ascend to that point where scientists lose all evidence of the material drug, the system, entirely uninfluenced by medicine, overcomes the disease by the sheer *vis medicatrix naturæ* alone. But if this argument is tenable, it proves too much for its advocates, and shows that the drug action, in the lower potencies, is manifested by its *killing*, instead of its curative properties, and demonstrates that drugs in any form should never be administered to the sick.

Neither of these positions can be maintained, and the only satisfactory explanation is the conclusion that every physician will come to who will give the subject years of candid trial and investigation. What this conclusion will be after such clinical experience there can be no doubt, and, without it, no one is competent to decide. The great cause of eclectic-homœopathy and heterogeneous prescribing is, first, in being so instructed by those who know no better; and secondly, in young prescribers relying on their own judgments, which, if not biased by prejudice, certainly can not be reliable guides. What is needed is more clinical instruction under the supervision of experienced prescribers. Young men hurry through college and commence practice with more knowledge of almost everything else than how to cure the sick; this they are obliged to find out by years of experimenting at the expense of their patients. In this way a routine habit of prescribing is established—a therapeutic groove, or rut is formed, out of which the physician may not for years, if ever, be able to extricate himself.

Every prescriber could easily become a legitimate Homœopathist with the proper clinical instruction, but as it now is we can only say in regard to very many of them, in the language of Dick Dead-Eye, "They mean well, but they don't know."

